# Image-based molecular representation learning for drug development: a survey

**DOI:** 10.1093/bib/bbae294

**Published:** 2024-06-26

**Authors:** Yue Li, Bingyan Liu, Jinyan Deng, Yi Guo, Hongbo Du

**Affiliations:** Division of Gastroenterology, Dongzhimen Hospital, Beijing University of Chinese Medicine, No. 5 Haiyun Warehouse, 100700, Beijing, China; School of Computer Science, Beijing University of Posts and Telecommunications, No.10 Xituchen Street, 100876, Beijing, China; Division of Gastroenterology, Dongzhimen Hospital, Beijing University of Chinese Medicine, No. 5 Haiyun Warehouse, 100700, Beijing, China; Division of Gastroenterology, Dongzhimen Hospital, Beijing University of Chinese Medicine, No. 5 Haiyun Warehouse, 100700, Beijing, China; Division of Gastroenterology, Dongzhimen Hospital, Beijing University of Chinese Medicine, No. 5 Haiyun Warehouse, 100700, Beijing, China; Institute of Liver Disease, Beijing University of Chinese Medicine, No. 5 Haiyun Warehouse, 100700, Beijing, China

**Keywords:** Drug development, Image-based Molecule representation, Computer vision

## Abstract

Artificial intelligence (AI) powered drug development has received remarkable attention in recent years. It addresses the limitations of traditional experimental methods that are costly and time-consuming. While there have been many surveys attempting to summarize related research, they only focus on general AI or specific aspects such as natural language processing and graph neural network. Considering the rapid advance on computer vision, using the molecular image to enable AI appears to be a more intuitive and effective approach since each chemical substance has a unique visual representation. In this paper, we provide the first survey on image-based molecular representation for drug development. The survey proposes a taxonomy based on the learning paradigms in computer vision and reviews a large number of corresponding papers, highlighting the contributions of molecular visual representation in drug development. Besides, we discuss the applications, limitations and future directions in the field. We hope this survey could offer valuable insight into the use of image-based molecular representation learning in the context of drug development.

## Introduction

The advances in biomedical technologies are inextricably linked to drug research and development [1], which is a time-consuming and money-consuming process that often takes 10–15 years with an investment of billions of dollars [2]. Typically this process encompasses several stages, including drug discovery, drug development, clinical trials and approval applications, during which we search for candidate compounds with therapeutic effects for specific diseases among hundreds or thousands of compounds [3] as well as ensuring the pharmacokinetics, efficacy and safety of candidate compounds. Due to the complexity, traditional experimental methods require enormous development cost and labor effort, making it hard to quickly evaluate all candidate compounds under a large-scale scenario.

**Figure 1:**
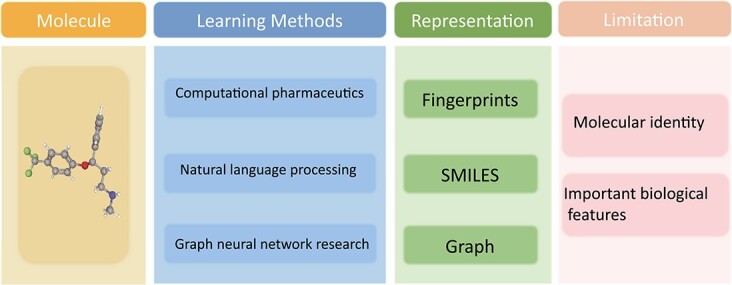
Commonly used methods for molecular representations in existing literature. All of them fail to achieve accurate molecular identity and feature extraction due to the intrinsic limitation of their learning principles, which motivates researchers to explore a new type of representation.

With the popularity of artificial intelligence (AI) [4], researchers have attempted to take advantage of AI technologies for accurate and fast drug development [5–7]. Specifically, they pay more attention to **molecule representation**, which is the base for the subsequent drug research. Traditional methods in this field can be classified into three categories: *computational pharmaceutics*, *natural language processing (NLP)* and *graph neural network (GNN)* research. Computational pharmaceutics involves representing molecules with fingerprints [8], topological indices [9] or substructure fragments [10], also known as descriptors [11]. Despite being widely adopted, these methods fail to reflect the explicit structural information of molecules. NLP approaches refer to representing molecules in a format like Simplified Molecular Input Line Entry System (SMILES) [12] or International Chemical Identifier (InChI) [13], such that we can treat them as a type of natural language and utilize some NLP methods for processing. GNN research focuses on representing molecules as graphs, typically using an adjacency matrix and processing them with GNNs [14] (e.g. MIT’s DMPNN [15], Alex *et al*. [16] and Cho *et al*. [17]). As shown in Fig. 1, although these approaches (i.e. NLP and GNN) offer potential advantages of AI technologies, they may have limited expressiveness in accurately capturing molecular identity and important biological features.

In recent years, the research community has begun to explore molecules in a form of images, as each chemical substance has a unique visual representation [18]. Using visual representation has the following advantages: (1) By analyzing the type of atoms, their relative positions and the connections between atoms in the image, different chemical substances can be effectively identified. Representing molecules using 2D molecular images offers simplicity and intuitiveness, making it a compelling choice for molecular design and analysis. (2) Computer vision (CV) based techniques have been successfully used to address multiple image related applications, such as object recognition, object detection and automatic driving [19–22], which indicates that there may be a variety of available image processing skills to benefit the effective molecular visual representation. (3) This type of method has the potential to capture and extract complex underlying structural patterns and features such as attribute relationships. In addition, the knowledge obtained from these dataset-specific descriptors can be used to better interpret and understand structure–property relationships and design new compounds, outperforming models trained with molecular descriptors and fingerprints.

Unfortunately, to the best of our knowledge, there is no existing survey that is specially focused on the image-based molecular representation. As shown in Table 1, current surveys either review the general AI techniques [18, 23–34] or dive into the specific techniques such as NLP and GNN [35–41] for drug development. For example, Deng *et al*. [34] provided a comprehensive review on the application of AI in drug discovery by dissecting AI technologies into model architectures and learning paradigms. Wieder *et al*. [39] conducted a comprehensive review by collecting and categorizing 80 GNNs, summarizing 20 molecular properties across 48 different datasets used for prediction. Considering the rapid research progress of molecular visual representation and CV, it is necessary and urgent to give a timely and specific survey to summarize and classify the vision related techniques of drug development. Here the novelty lie in the perspective of our survey (i.e. summarizing image-based learning techniques for drug development).

**Table 1:** Current surveys on molecular representation learning for drug development. As shown in the table, all of them focus on GAIT or specific techniques such as NLP and GNN, leaving the image-related learning models unexplored

		Specific techniques	
Ref.studies	GAIT	NLP	GNN
Chen, 2018 [23]	$\checkmark $		
Vamathevan, 2019 [24]	$\checkmark $		
Elton, 2019 [25]	$\checkmark $		
Xu, 2019 [26]	$\checkmark $		
Wieder, 2020 [39]			$\checkmark $
Ozturk, 2020 [36]		$\checkmark $	
Brown, 2020 [27]	$\checkmark $		
Mercado, 2020 [38]			$\checkmark $
Chuang, 2020 [28]	$\checkmark $		
Sun, 2020 [37]			$\checkmark $
Jiménez, 2021 [29]	$\checkmark $		
Paul, 2021 [30]	$\checkmark $		
Xiong, 2021 [40]			$\checkmark $
Meyers, 2021 [18]	$\checkmark $		
Sousa, 2021 [31]	$\checkmark $		
Kumar, 2021 [32]	$\checkmark $		
Blay, 2022 [33]	$\checkmark $		
Deng, 2022 [34]	$\checkmark $		

GAIT: General AI techniques.

In this paper, we provide a comprehensive survey on CV-based molecular representation, targeted at reviewing the recent advanced visual methods designed for medicinal molecule research. Concretely, our survey takes the learning paradigms in the area of AI as the key guidance, and proposes a taxonomy in terms of how to utilize images to conduct different learning paradigms in the context of drug development, where we briefly introduce some representative works. In conclusion, the key contributions of this survey are as follows.

- We investigate how the molecular visual representation contributes to the drug development. To the best of our knowledge, this is the first survey that specifically concentrates on image-based molecular representation learning.
- We propose a new taxonomy based on image-related learning paradigms, and review a large number of research papers to thoroughly summarize the advance in the field of drug development.
- We overview the image-empowered applications of drug development and discuss some potential deficiencies and future directions.

The remainder of this survey is structured as follows. In Section ‘Preliminaries’, we first introduce preliminaries of molecular visual representation, including the data preparation and the pipeline of molecular image processing. In Section ‘Image-based Learning Paradigms for Drug Development’, we propose the taxonomy of CV-based molecular representation learning, in which various CV approaches are discussed and categorized. Then, in Section ‘Applications’, we introduce some prevalent applications to show the practical usage of molecular visual representation. Finally, Section ‘Limitations’ and Section ‘Conclusion’ discuss the future work and conclude this paper.

## Preliminaries

In this section, we provide an overview of how to represent molecules in an image-based model and introduce several publicly available data resources in this field.

### Molecule visual representation

**Figure 2:**
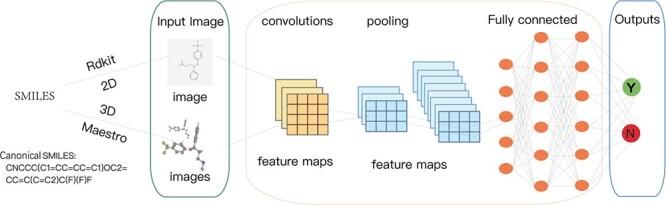
A general pipeline of molecule visual representation. Here we use the CNN as an example. It mainly includes four steps: (1) extracting the compounds in a SMILES format from datasets; (2) converting the molecules represented in SMILES format into images; (3) feeding the image representation of the molecule into a CNN model; (4) using optimization algorithms to train the CNN model to make predictions and analysis.

In order to obtain the molecule visual representation, it is necessary to convert compound molecules in an image format. Here we take convolutional neural network (CNN), the typical CV model, as an example to illustrate the pipeline. As shown in Fig. 2, this process includes the following steps:

- *Step1: Extracting the compounds in a SMILES format from datasets.* SMILES [12, 42, 43] is a widely used chemical notation system for representing molecular structures. It is commonly used in various databases as a query format for drugs or drug-like compounds. We first extract the data in a SMILES format so that they can be effectively processed for the subsequent steps.
- *Step2: Converting the molecules represented in SMILES format into images.* The open-source tool RDKit [44, 45] employs a series of algorithms and computations to achieve this conversion. It parses the SMILES string by creating an internal representation called a molecular graph. RDKit then generates a set of molecular conformers, representing different spatial arrangements of the molecule. Using selected conformers, RDKit further applies algorithms to generate a 2D layout of the molecule, taking into account the specific conformations. This layout is then rendered as an image, and customization options allow for adjustments to the image size and other visual aspects. For 3D images, the dataset is imported into Maestro [46], and each 3D conformation is globally rotated eight times around the y-axis, with each rotation being $45^{\circ }$ to capture the image from different angles. In Maestro, global rotation does not affect the actual molecule coordinates, and the image of the current view is saved after each rotation.
- *Step3: Feeding the image representation of the molecule into a CNN.* Given a molecule image, the CNN model will extract and learn features of the molecule through a series of convolutional, pooling and fully connected layers. Each layer of CNN transforms the input quantity into the output quantity of neuron activation, eventually leading to the final fully connected layer, which maps the input data to a one-dimensional feature vector.
- *Step4: Using optimization algorithms to train the CNN model to make predictions and analysis.* After obtaining the outputs from the last step, we finally employ some optimization algorithms to train the weights of the CNN model, such that the model can predict properties, activity, interactions or other relevant features of the molecules.

### Public databases

As shown in Table 2, we list several commonly used public databases in the community, which can be briefly summarized as follows.

**Table 2:** Several public databases used for drug development. Here we provide the name, brief description, url and references of these databases, aiming at facilitating related research

Database name	Description	URL	References
PubChem	PubChem, managed by the National Library of Medicine (NLM) under the National Institutes of Health (NIH) in the United States, is a leading public chemical database, that offers open access and draws data from 750+ sources.	https://pubchem.ncbi.nlm.nih.gov/	[47, 48]
ChEMBL	ChEMBL, encompassing a wealth of bioactive molecules possessing drug-like attributes, is a leading repository. Its comprehensive framework seamlessly integrates chemical, bioactivity, and genomic data.	https://www.ebi.ac.uk/chembl/	[49, 50]
ZINC	ZINC, a collaborative compound repository from UCSF’s Irwin and Shoichet labs, concentrates on diverse compound structures, featuring an intuitive search interface.	https://zinc.docking.org/	[64, 51]
DrugBank	DrugBank, a dynamic online resource, provides detailed insights into FDA-approved drugs and investigational compounds.	https://go.drugbank.com/	[52, 53]
SIDER	SIDER captures information on drugs that have successfully reached the market. Drawing from public documents and parsed package inserts, it offers data on the frequency and classification of side effects, along with crucial details on drug-target interactions.	http://sideeffects.embl.de/about/	[54, 55]
OFFSIDES TWO-SIDES	OFFSIDES captures drug side effects that have been discovered but are absent from FDA labels. TWO-SIDES meticulously maps drug-drug interactions and their effects, shedding light on the complex interplay between medications and the human body.	https://tatonettilab.org/offsides/	[56, 57]
DILIrank	DILIrank is a substantial database focused on drug-induced liver toxicity.	https://www.fda.gov/science-research/liver-toxicity-knowledge-base-ltkb/drug-induced-liver-injury-rank∖-dilirank-dataset	[58, 59]
LiverTox	LiverTox, developed by the National Institute of Diabetes and Digestive and Kidney Diseases (NIDDK), is a contemporary resource that unravels liver injury caused by substances, including prescription drugs.	https://www.ncbi.nlm.nih.gov/books/NBK547852/	[60, 61]
DILIst	DILIst employs statistical methodologies to categorize drugs into DILI-positive and DILI-negative groups. Notably, DILIst demonstrates a 65% increase in classification compared to the pioneering DILIrank.	https://www.fda.gov/science-research/liver-toxicity-knowledge-base-ltkb/drug-induced-liver-injury-severity∖-and-toxicity-dilist-dataset	[62, 63]

From the table, we can see that several commonly used large public databases are available, such as PubChem [47, 48], ChEMBL [49, 50] and ZINC [51, 64]. These databases contain a significant amount of chemical structures and activity data points determined in biological laboratories and offer online queries and downloads. Public databases related to marketed drugs and their effects on humans, such as DrugBank [52, 53], SIDER [54, 55], OFFSIDES and TWO-SIDES [56, 57], DILIrank [58, 59], livertox [60, 61] and DILIst [62, 63], are also available. Researchers can easily acquire related data for their AI model construction.

To evaluate the performance of molecule property prediction and molecule generation, Wu *et al*. [65] released MoleculeNet in 2018, which includes a set of datasets and an open-source DeepChem package. This benchmark dataset covers four categories: quantum mechanics (QM7, QM7b, QM8, QM9), physical chemistry (ESOL, FreeSolv, lipophilicity), biophysics (PCBA, MUV, HIV, PDDBind, BACE) and physiology (BBBP, Tox21, ToxCast, SIDER, ClinTox), involving single or multitask. It is important to note that datasets for molecule property prediction may be highly imbalanced, and medicine-related databases may have limited data and slow updates. 

## Image-based learning paradigms for drug development

In this paper, we propose a novel taxonomy to categorize and summarize literature in terms of the image-based learning paradigms. In order to facilitate a better understanding of image-based AI models for the readers, we first provide a brief introduction to the knowledge of CNNs, which are considered as the typical architecture in the field of CV.

The CNN [66] is inspired by the structure of the visual system and is a computational model that describes a form of obtaining translational invariance when applying neurons with the same parameters to blocks of the previous layer at different positions. CNNs consist of three main types of neural layers: convolutional layers, pooling layers and fully connected layers, with each type of layer playing a different role.

The convolutional layers perform the convolution operation on the input data, utilizing filters (also known as kernels) with weights and biases to extract features. The convolution operation can be represented as 


(1) \begin{align*}& \mathbf{Y} = \mathbf{X} * \mathbf{K},\end{align*}

where $ \mathbf{Y} $ is the output feature map, $ \mathbf{X} $ is the input feature map and $ \mathbf{K} $ is the set of convolutional kernels. The convolution operation in the context of deep learning for a 2D input can be represented as 


(2) \begin{align*}& (F * G)(i, j) = \sum_{m=0}^{M-1} \sum_{n=0}^{N-1} F(i-m, j-n) \cdot G(m, n),\end{align*}

where $ F $ represents the input feature map, $ G $ the convolution kernel and $ i, j $ the spatial indices of the output feature map. $M$ and $N$ denote the height and width of the convolution kernel.

Pooling layers reduce the spatial dimensions of the feature maps obtained from the convolutional layers while retaining important features. Common pooling operations include max pooling and average pooling.

The fully connected layers connect every neuron in the previous layer to the neurons in the subsequent layer. They map the features learned from the previous layers to a one-dimensional feature vector. The fully connected layers can be expressed as 


(3) \begin{align*}& y = f(Wx + b),\end{align*}

where $ y $ is the output vector, $ W $ is the weight matrix, $ x $ is the input vector, $ b $ is the bias vector and $ f $ is the activation function. These distinctions ensure that the unique characteristics of each operation are clearly represented.

**Figure 3:**
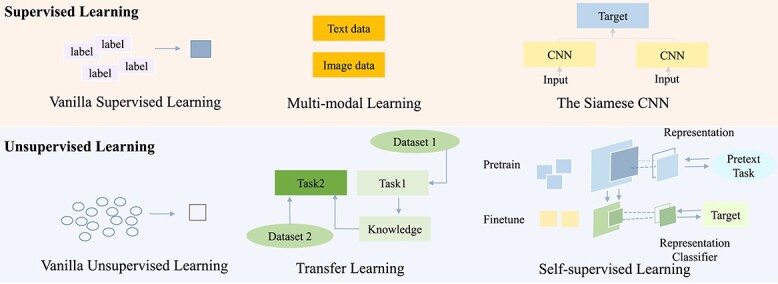
Illustration of various learning paradigms for image-based processing. Specifically, supervised/unsupervised learning refers to optimizing AI models with labeled/unlabeled data. Multi-modal learning means that we use different data modals (e.g. text, image) to collaboratively optimize AI models and the siamese CNN leverages contrastive learning to measure the similarity between two different inputs for more efficient learning. Transfer learning, instead, utilizes the knowledge from another similar task to assist the target task that lacks labeled data. Self-supervised learning takes advantage of the data property itself to generate related labels for optimization.

**Figure 4:**
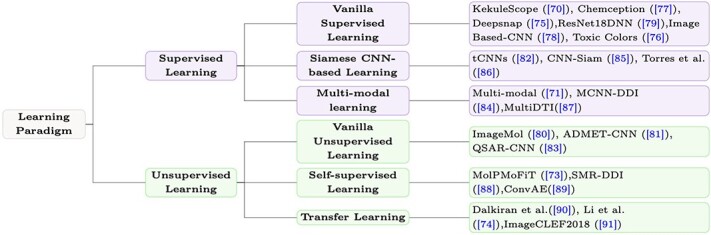
Taxonomy based on different learning paradigms. Here we list the representative paradigms and their corresponding works. We would like to clarify that ImageCLEF2018 is the name of the proposed method. The date of ’2018’ is not specifically added by us. Besides, we notice that some of works are not given a specific name in their original papers and thus we adopt the form of ‘XX *et al*.’ to denote the work.

In the context of molecular characterization and molecular property prediction, labeled data points are essential for machine learning models [67]. However, generating labeled data points in a laboratory setting is typically costly. As a result, the dataset used for model training is usually limited in size, sparse and may suffer from bias and noise, commonly known as the low-data drug discovery problem [68]. To overcome these challenges, various learning paradigms have been proposed [69]. As shown in Fig. 3, this survey primarily focuses on **supervised learning** and **unsupervised learning**, each of which includes several specific learning paradigms. In addition, the specific learning paradigms and corresponding works are summarized in Fig. 4. The illustration of different image-related models are recorded in Table 3 and a comprehensive list of tools/algorithms/codes/scripts for image-based molecular representation learning is given in Table 4.

**Table 3:** Illustration of different image-related models. We group the methods that use the same dataset and annotate each with the publication year, journal and specific citation to make the table clear. Note that it is impossible to align them on the same dataset for performance comparison since these methods employ different data selection schemes to evaluate their effectiveness in their original papers

Model	Database	Year	Published Journal	Ref. studies
KekuleScope	ChEMBL	2019	Journal of cheminformatics	[70]
Multi-modal	ChEMBL	2022	Briefings in Bioinformatics	[71]
DEEPScreen	ChEMBL,DrugBank	2020	Bioinformatics	[72]
MolPMoFiT	ChEMBL,MoleculeNet	2020	Journal of cheminformatics	[73]
Li *et al*.	ChEMBL29	2023	Journal of Innovative Optical Health Sciences	[74]
Deepsnap	Tox21	2020	Molecules	[75]
Toxic Colors	Tox21	2018	Journal of Chemical Information and Modeling	[76]
Chemception	HIV,Tox	2017	arXiv	[77]
Image-based CNN	Mulliner,Tox	2020	Journal of Chemical Information and Modeling	[78]
ResNet18DNN	DILIrank,livertox,etc	2022	Briefings in Bioinformatics	[79]
ImageMol	MoleculeNet,PubChem	2022	Nature Machine Intelligence	[80]
ADMET-CNN	PubChem	2019	Chemometrics and Intelligent Laboratory Systems	[81]
tCNNs	GDSC	2019	BMC bioinformatics	[82]
QSAR-CNN	literature	2021	Chemical Engineering Journal	[83]
MCNN-DDI	DrugBank,etc	2024	Scientific Reports	[84]

**Table 4:** A comprehensive list of tools/algorithms/codes/scripts for image-based molecular representation learning. For the algorithms that are not given a specific name, we use XXX *et al*. to denote them

**Learning Paradigm**	**Specific Usage**	**Tools/Algorithms**	**Codes/Scripts(url)**
Molecule Visual Representation	2D Image Processing	RDkit	https://github.com/rdkit/rdkit
	3D Image Processing	Maestro	https://github.com/maestro-project/maestro
Supervised Learning	Vanilla Supervised Learning	Kekulescope	https://github.com/isidroc/kekulescope
		Chemception	https://github.com/Abdulk084/Chemception
		Deepsnap	https://github.com/snap-stanford/deepsnap
	Siamese CNN-based Learning	tCNN	https://github.com/Lowpassfilter/tCNNS-Project
	Multi-modal learning	MultiDTI	https://github.com/Deshan-Zhou/MultiDTI
Unsupervised Learning	Vanilla Unsupervised Learning	ImageMol	https://github.com/HongxinXiang/ImageMol
	Self-supervised Learning	MolPMoFiT	https://github.com/XinhaoLi74/MolPMoFiT
		convAE	https://github.com/dmitrav/pheno-ml
	Transfer Learning	Dalkiran *et al*.	https://github.com/cansyl/TransferLearning4DTI

### Supervised learning

Supervised learning [85] trains a model using labeled data to establish a predictive relationship between input data and their respective labels. This learning paradigm is highly accurate in predicting outcomes, with the added benefit of convenient feature extraction and easily verifiable results. Consequently, supervised learning can be applied in fields such as molecular characterization, molecular property prediction and toxicity prediction. For instance, it has played a crucial role in drug discovery, where accurate predictions of molecular properties and toxicity are essential in identifying potential drug candidates and understanding their effects on biological systems. In the following parts, we describe some typical supervised learning paradigms, including vanilla supervised learning, siamese CNN-based learning and multi-modal learning.

#### Vanilla supervised learning

Vanilla supervised learning means that we just conduct typical supervised learning for drug development. For instance, KekuleScope [70] is a model that has showcased comparable performance with circular fingerprint-based RF and DNN models, all while bypassing the need for intricate composite descriptors or advanced image processing techniques. It achieves this using a dataset of 33 IC50 values extracted from ChEMBL 23. By building upon existing architectures (such as AlexNet, DenseNet-201, ResNet152 and VGG-19), KekuleScope accurately forecasts the *in vitro* activity of compounds against cancer cell lines and protein targets, relying solely on the inherent Kekulé structures. Notably, these architectures were initially pre-trained on disparate image datasets.

Chemception [77], another model, using just the images of 2D drawings of molecules without providing any additional explicit chemistry knowledge, such as basic concepts like periodicity, or advanced features like molecular descriptors and fingerprints, has shown slightly better performance in predicting biochemical activity and solvation compared with Quantitative Structure-Activity Relationship (QSAR) models [86] based on molecular fingerprints, but slightly inferior performance in predicting toxicity. 3D images are also useful in QSAR modeling, and the Deepsnap [75] approach enables adjustment of parameters such as atom, atom color, bond radius and pixel size in the process of building 3D chemical structures. This approach has higher predictive performance, providing detailed chemical structure information from different viewing angles, which can reveal key toxic conformations of chemical substances and protein structure domains related to biological activity.

For toxicity prediction, graph-based CNN models and molecular images have been used to construct molecular toxicity classification models [76, 78]. Fernandez *et al*. put forward a proposition where they accomplished Tox21 benchmark predictions employing fundamental two-dimensional chemical sketches, entirely excluding the utilization of any chemical descriptors. Notably, they employed a supervised 2D CNN (2DConvNet) to handle uncomplicated molecular 2D sketches. Their findings underscore that the predictive precision attained through contemporary image recognition techniques can stand on par with the capabilities of state-of-the-art chemoinformatics tools. Upsampling techniques such as the COVER method have been used to address the problem of small data size in toxicity databases. To identify and address potentially problematic drug candidates in early stages, Asilar *et al*. [78] employed 3D conformational images with a CNN, connecting chemical features with compound geometry. Additionally, they apply the COVER method for dataset upsampling and class balance. Validated on Tox21 data, results align with challenge winners, encouraging liver toxicity prediction. Using a comprehensive public liver toxicity dataset, they achieve 0.79 sensitivity and 0.52 specificity, confirming the viability of image-based toxicity prediction with deep neural networks. Additionally, Zhao *et al*. [79] proposed using an 18-layer residual neural network with more five-layer blocks (ResNet18) and a deep neural network (ResNet18DNN) model to predict drug-induced liver injury, achieving the highest prediction accuracy to date.

**Discussion.** Supervised learning leverages labeled data to train models, efficiently extracting valuable patterns and insights from historical data. This allows for effective data utilization. With a large amount of high-quality labeled data, it can provide highly accurate predictions. Its applications are widespread, and the models often possess good interpretability. However, supervised learning heavily relies on high-quality labeled data, which can be costly and time-consuming to obtain in drug development. If the training data has biases, such as data imbalance or labeling errors, the model’s predictions can be significantly affected, potentially leading to misleading results and poor decision-making in drug development. Moreover, the model’s generalization ability is limited, which is particularly evident in the complex and variable environment of drug research. The lack of deep understanding of drug mechanisms means the model may only capture superficial data patterns. In medical scenarios with limited data or low data quality, overfitting is a common issue. 

#### Siamese CNN-based learning

The Siamese Convolutional Neural Network model [87] is a special type of CNN model that is commonly used for similarity measurement or matching tasks between two inputs. The structure of the Siamese model includes two identical branches of the CNN, each processing one input, and then their outputs are compared and fused to output a similarity measurement or classification result. The Siamese CNN can handle input data of different sizes, and the two identical CNN branches share parameters, so the input data can be converted into the same size feature vector for comparison. It has a certain tolerance for noise and deformation in the input data, demonstrating strong robustness. Training Siamese CNNs requires defining a loss function that measures how well the model’s predictions match the actual labels for similar and dissimilar samples. The commonly used loss function is called Contrastive Loss. The formula for the Contrastive Loss function is as follows: 


(4) \begin{align*}& \mathcal{L}(W, I_ {1}, I_ {2} )=1(L=0) \frac{1}{2} D^ {2} +1(L=1) \frac{1}{2} [\max (0,margin-D)]^ {2},\end{align*}

where the input consists of a pair of retina fundus images, denoted as $I_{1}$ and $I_{2}$, which are independently fed into two identical CNNs. The indicator function $1(\cdot )$ is employed to determine whether the two images share the same label. When $L = 0$, it indicates that the images have the same label, whereas $L = 1$ signifies the opposite case. The parameter vector $W$ is shared across the neural networks and learned during training. The latent representation vectors of $I_{1}$ and $I_{2}$ are denoted as $f(I_{1})$ and $f(I_{2})$, respectively. The Euclidean distance $D$ between $f(I_{1})$ and $f(I_{2})$ is computed as $\|f(I_{1}) - f(I_{2})\|_{2}$.The model is suitable for various similarity tasks, such as image retrieval [88], face recognition [89] and speech recognition [90]. By comparing the similarity of the two feature vectors in the last layer, different types of similarity tasks can be completed.

**Figure 5:**
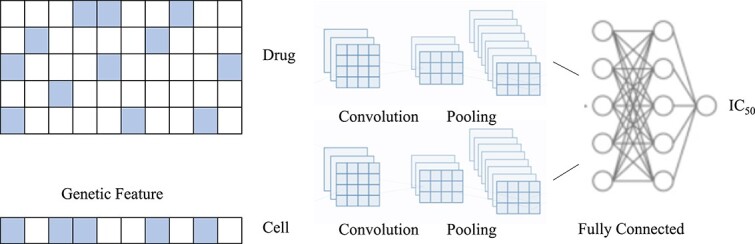
Illustration of the twin CNNs model. The key idea is to utilize a convolutional network to extract drug features from molecules and another convolutional network to extract features of cancer cell lines from genetic feature vectors.

As shown in Fig. 5, Liu *et al*. [82] proposed a typical twin convolutional neural networks (tCNNs) model for phenotype screening, which utilizes a convolutional network to extract drug features from molecules and another convolutional network to extract features of cancer cell lines from genetic feature vectors. Subsequently, a fully connected network is used to predict the interactions between drugs and cancer cell lines. The performance of this model is significantly superior to previous studies, and it can be applied to small training datasets and a reduced number of cancer cell line features, making it cost-effective. Yang *et al*. [91] proposed a deep learning-based model that utilizes a dual CNN to learn representations of multi-modal drug data and predict potential drug response types. Torres *et al*.[92] explored a siamese neural network architecture for one-shot drug discovery.

**Discussion.** Siamese CNNs demonstrate numerous advantages in drug development, particularly in handling small sample sizes and metric learning tasks. They are highly effective for addressing small sample problems, calculating molecular similarities and drug repositioning. By comparing the similarities in molecular structures or properties, they can predict new potential drugs. Additionally, they require relatively less labeled data, alleviating the pressure of obtaining high-quality labeled data in drug research. It is worth noting that, due to the need to train two networks, it requires strong computational resources and professional knowledge and experience. Integrating other methods and improved techniques can help mitigate these drawbacks and enhance their effectiveness in practical use.

#### Multi-modal learning

Multi-modal learning [93] aims to learn knowledge from multiple modalities, such as images, speech and text. It integrates information from different modalities into one model to obtain more comprehensive and accurate results than a single modality. Multi-modal learning integrates data from diverse sources (images and text) to construct a joint representation, denoted as $X_{\textrm{joint}}$, effectively capturing complementary information. The learning model takes this joint representation as input and performs predictions by minimizing a loss function L during training, where the model parameters are optimized to minimize the difference between its predictions and the true labels Y. This approach enhances performance in tasks such as classification or regression, as it efficiently combines information from multiple modalities.

Molecular structure images contain rich structural information in visual form (compounds and molecular formulas), and image processing algorithms have been widely used to extract this information. However, the low quality and high noise of images affect the accuracy of obtaining molecular structures. Therefore, combining images with text information seems to be able to improve the model accuracy and obtain higher robustness and better generalization ability.

**Figure 6:**
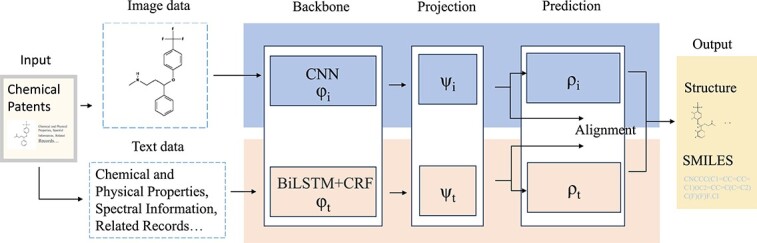
Illustration of multi-modal learning on drug development. The core idea is to develop an AI model to simultaneously cope with different data modals (i.e. image and text) for drug recognition.

As shown in Fig. 6, Wang *et al*. [71] proposed a multi-modal chemical information reconstruction system that can automatically process, extract and align heterogeneity information from text descriptions and structural images of chemical patents. The key innovation is the heterogeneity data generator, which generates cross-modal training data in the form of text descriptions and Marsh structure images, and then a dual-branch model with image and text processing units can learn to identify heterogeneous chemical entities and capture their corresponding relationships. Based on the reconstruction results and substitution rules, a large-scale library of near-pharmaceutical compounds can be automatically generated. In quantitative evaluations, the model can correctly reconstruct 97% of molecular images into structured formats and achieve an F1 score of about 97–98% in chemical entity recognition. MCNN-DDI [84] and MultiDTI [94] have achieved superior accuracy compared with traditional prediction algorithms by employing multi-modal training through inputs such as chemical structure (i.e. drug smiles), enzymes, pathways and drug targets. It should be noted that multi-modal data need to be collected and integrated from different sources. On the one hand, whether text and image data are of high quality will directly affect the model’s generalization ability. On the other hand, there may be data imbalance issues between different modalities, which may cause the model to learn insufficiently or over-learn certain modalities. In addition, the learning effect of multi-modal learning is difficult to quantify and evaluate, as the contribution between different modalities is difficult to accurately measure.

**Discussion.** Multimodal learning offers significant advantages in drug development by integrating various types of data to enhance predictive accuracy and model robustness. This comprehensive approach provides a more holistic perspective, aiding in more precise drug discovery and development while uncovering potential biological associations. These insights are crucial for understanding drug mechanisms and adverse effects. However, challenges in data integration, computational costs, model design and standardization cannot be overlooked. Currently, the application of multimodal learning in drug development is not yet mature, lacking standardized methods and tools, which makes it difficult to compare and reproduce results across different studies. Additionally, the heavy reliance on data quality is a major issue, as poor-quality data from any single modality can degrade overall model performance. The varying costs and difficulties associated with acquiring different types of data further complicate practical applications. By continuously improving data processing methods and model design, the potential of multimodal learning in drug development can be further realized.

### Unsupervised learning

Unsupervised learning [95] is a machine learning technique that automatically discovers patterns and structures in unlabeled data. Unlike supervised learning, unsupervised learning does not require labeled data, making it more convenient to use massive amounts of unlabeled data for exploring the underlying structure and patterns in the data. This helps to understand the intrinsic rules and characteristics of the data. When combined with NLP and unsupervised learning methods, such as SMILES, InChI or two-dimensional graphics, CV and unsupervised pre-training models can improve the accuracy of information vectors describing molecular identity and biological features. By leveraging large-scale unlabeled molecular data, unsupervised models can learn valuable molecular representations, which can be fine-tuned for specific tasks like drug toxicity prediction or protein-ligand binding affinity estimation. This transfer learning approach enables the extraction of intricate molecular features that generalize well to various downstream tasks. However, considering that unsupervised learning lacks strong supervision information, it fails to predict accurately as supervised learning. Furthermore, the lack of labeled data makes it difficult to verify and accurately evaluate the model’s performance. Therefore, unsupervised learning is better suited as a pre-trained base for downstream tasks. In the following parts, we briefly introduce some specific unsupervised learning paradigms. 

#### Vanilla unsupervised learning

There are a large number of works that contribute to drug development using vanilla unsupervised learning. ImageMol [80], for example, has chemical awareness and can learn molecular structure from large-scale molecular images. Using molecular images as feature representations of compounds, it has high accuracy and low computational cost. It also uses an unsupervised pre-training framework to capture the structural information of molecular images from 10 million drug-like compounds with different biological activities in the human proteome. To ensure the maximum correlation between molecular structures in the image, the model performs the following processing on the input images. First, the latent feature $ f_{\theta }(x_{n}) $ is reconstructed into a $64\times 64$ molecular image $ G(F_{\theta }(X_{n})) $. The original $224\times 224$ molecular image$x_{n}$ is then resized to 64$\times $64 and input together with the molecular images generated by G into the discriminator D, obtaining $D( x_{n}^{64 \times 64})$ and $ D(G(F_{\theta }(X_{n}))) $. Finally, they update the parameters of the generator G and discriminator D using the cost function, where $ \mathcal{L}_{G} $ and $ \mathcal{L}_{D} $ are defined as follows: 


(5) \begin{align*} & \mathcal{L}_{G} = E[D(G(f_{0}(x_{n})))] + ||G(f_{\theta}(x_{n})), x_{n}^{64 \times 64}||_{2} \end{align*}

(6) \begin{align*} & \mathcal{L}_{D} = E[D(x_{n}^{64 \times 64})] - E[D(G(f_{\theta}(x_{n})))]\end{align*}

For $ \mathcal{L}_{G} $, the first term represents the Wasserstein loss, and the second term measures the Euclidean distance between the generated image $ G(f_{\theta }(x_{n})) $ and the corresponding real image $x_{n}^{64 \times 64}$. For $ \mathcal{L}_{D} $, we use this loss to approximate the Wasserstein distance between the real image distribution $x_{n}^{64 \times 64}$ and the fake image distribution $ G(F_{\theta }(X_{n})) $. Finally, the molecular encoder model $ \mathcal{L}_{MIR} $ is updated using the cost function, formalized as


(7) \begin{align*} \mathcal{L}_{MIR} = E[D(G(f_{\theta}(x_{n})))] + ||G(f_{\theta}(x_{n})), x_{n}^{64\times 64} ||_{2} - E[D(G(f_{\theta}(x_{n})))]\end{align*}

As reported in the corresponding paper, its performance is better than Chemception [77], ADMET-CNN [81] and QSAR-CNN [83].

**Discussion.** Vanilla unsupervised learning offers certain advantages in drug development. On the one hand, it can reveal hidden patterns and structures within complex biological data without predefined labels, such as the identification of new drug targets and biomarkers. Techniques like clustering and dimensionality reduction help summarize large databases, making it easier to visualize and interpret complex relationships within the data. Additionally, unsupervised learning does not require the scarce and expensive labeled data often needed in drug development, allowing for the utilization of vast amounts of unlabeled biological and chemical data. By exploring data without bias, unsupervised learning can also generate new hypotheses and insights, guiding further experimental studies and drug discovery efforts. However, despite these advantages, there are notable drawbacks. Unsupervised learning models do not provide clear explanations for the patterns they discover, making the results difficult to interpret. This can hinder the understanding of underlying biological mechanisms and specific drug development outcomes, requiring additional steps and validation to translate findings into practical applications. Furthermore, unsupervised learning is highly sensitive to the quality and preprocessing of input data; noise and irrelevant features can significantly affect results, leading to misleading conclusions. The scalability of these methods also presents challenges, as processing large-scale biological data requires substantial computational resources. Lastly, the lack of standardized methods and evaluation criteria in unsupervised learning makes it difficult to compare and replicate results across different studies. By addressing these challenges through improved algorithms, data preprocessing techniques, and integration with other methods, the potential of vanilla unsupervised learning in drug development can be further enhanced. 

#### Self-supervised learning

Self-supervised learning [96] is a machine learning method that utilizes the characteristics of data to automatically generate labels or targets, and then trains the model through supervised learning to ultimately obtain a model that can solve a specific task. In contrast to supervised learning, which requires explicit labels or targets, self-supervised learning generates implicit labels or targets on its own and has achieved state-of-the-art performance in limited label learning. Although self-supervised learning falls under unsupervised learning, it should be differentiated from unsupervised learning, which focuses on detecting patterns in unlabeled data, such as clustering, while self-supervised learning aims to recover data. Specifically, self-supervised learning can be divided into two main types: generative and contrastive self-supervised learning. Here are some common formulas used in self-supervised learning:

*Autoencoder*: Autoencoders are the most common self-supervised learning method, aiming to reconstruct the input data. The autoencoder can be represented as 


(8) \begin{align*} & Encoder (f_{\textrm{enc}}): z = f_{\textrm{enc}}(x) \end{align*}

(9) \begin{align*} & Decoder (f_{\textrm{dec}}): \hat{x} = f_{\textrm{dec}}(z),\end{align*}

where $x$ represents the input data, $z$ is the encoded feature representation and $\hat{x}$ is the reconstructed output from the decoder. The encoder and decoder parameters are trained by minimizing the reconstruction error.

*Contrastive Learning*: Contrastive learning is a method that learns feature representations by comparing different views of the same sample. The commonly used contrastive loss function is the Negative Contrastive Loss: 


(10) \begin{align*}& \mathcal{L}_{\textrm{neg}}(x, x^{+}) = -\log \frac{\exp(f(x) \cdot f(x^{+}))}{\sum_{x_{i} \in \mathcal{X}} \exp(f(x) \cdot f(x_{i}))},\end{align*}

where $x$ represents the sample, $x^{+}$ represents a positive sample (from the same class or a different view of the same sample) and $f(cdot)$ represents the feature extraction function.

*Generative Models-based Methods*: Self-supervised learning can also be achieved through generative models, including autoregressive models and generative adversarial networks (GANs) [97]. Autoregressive models are a class of generative models where the model predicts the next element in a sequence based on the previous elements. Mathematically, an autoregressive model predicts the probability distribution of the next element $x_{t}$ given the previous elements $x_{1:t-1}$:


(11) \begin{align*}& P(x_{t} | x_{1:t-1})\end{align*}

GANs consist of a generator and a discriminator network. The generator aims to generate data that are indistinguishable from real data, while the discriminator’s task is to differentiate between real and generated data. The training of the GAN involves a minimax game between the generator and discriminator. The objective is to find the optimal parameters for both networks that minimize the following value function: 


(12) \begin{align*} \min_{G} \max_{D} V(D, G) =&\ \mathbb{E}_{x \sim p_{\textrm{data}}(x)} [\log D(x)] \nonumber\\ & +\mathbb{E}_{z \sim p_{z}(z)} [\log(1 - D(G(z)))],\end{align*}

where $V(D, G)$ is the value function. $p_{\textrm{data}}(x)$ is the true data distribution. $p_{z}(z)$ is the noise distribution from which $z$ is sampled. $\mathbb{E}$ represents the expectation.

Above mentioned are some common formula representations used in self-supervised learning and the specific self-supervised learning methods and models may vary depending on the task and data type. For the biomedical application, researchers proposed MolPMoFiT [73], an effective transfer learning method based on self-supervised pre-training and task-specific fine-tuning. This method was evaluated on four benchmark datasets (lipophilicity, FreeSolv, HIV and blood-brain barrier penetration) and showed the best performance. Kpanou *et al*. [98] introduced SMR-DDI, a self-supervised framework that employs contrastive learning to embed drugs into a scaffold-based feature space. This approach enables the identification of potentially hazardous drug combinations using solely structural information. Dmitrenko *et al*.[99] proposed two novel methods for utilizing imaging data to study the temporal and morphological phenotypic effects induced by different experimental conditions, applying them to analyze the effects of drugs in 2D cancer cell cultures.

**Discussion.** Similar to traditional unsupervised learning, self-supervised learning can efficiently utilize unlabeled data. Additionally, self-supervised learning excels at learning complex and robust features by predicting parts of the data from other parts, which is beneficial for downstream tasks such as target interaction and toxicity prediction. Models pre-trained with self-supervised learning also offer transferability, making them adaptable to various tasks with limited labeled data. Moreover, self-supervised learning can create multiple pretext tasks, such as predicting masked parts of molecular graphs or sequences, enabling a comprehensive understanding of drug development-related data. However, self-supervised models also face challenges, including model complexity, high computational demands, interpretability issues and sensitivity to data quality. 

#### Transfer learning

Transfer learning [100] involves utilizing knowledge gained in one domain to improve learning in another domain. This approach accelerates learning and enhances model performance by transferring a model’s learned results from one domain to another. It is a rapidly emerging technique that involves reusing pre-trained models built on large datasets as a starting point to create new and more optimized models for the target endpoint of interest, which is formalized as 


(13) \begin{align*}& y = f_{\text{pre-trained}}^{*}(h),\end{align*}

where $ h $ represents the extracted feature representation from the source model, $ y $ is the prediction result of the target model and $ f_{\text{pre-trained}}^{*} $ represents the optimized model on the top of the pre-trained model.

Specifically, the optimized model $ f_{\text{pre-trained}}^{*} $ is generated by fine-tuning, which involves adjusting the weights of the pre-trained model using the new task’s data. We can represent it as follows: 


(14) \begin{align*}& \theta^{\prime} = \theta - \eta \nabla_{\theta} \mathcal{L}(\theta; \mathbf{x}, y),\end{align*}

where $ \theta $ are the parameters of the pre-trained model, $ \eta $ is the learning rate, $ \mathcal{L} $ is the loss function, $ \mathbf{x} $ is the input data and $ y $ is the true label.

The typical transfer learning pipeline encompasses the following sequential stages: (1) Pretrained Model Selection: Choosing an appropriate pretrained model. (2) Data Preparation: Gathering data for both the source task and target task. (3) Model Modification: Extracting features and introducing new layers tailored to the target task. (4) Freezing Pretrained Layers: Preserving learned knowledge by locking certain layers. (5) Training: Adapting the model to the target task, with optional fine-tuning. (6) Regularization and Optimization: Mitigating overfitting with effective optimization techniques. (7) Evaluation and Iteration: Assessing model performance on a validation set, refining hyperparameters as needed.(8) Testing and Deployment: Validating the model on a separate test set before deploying. Adjust the pipeline based on task specifics.

**Figure 7:**
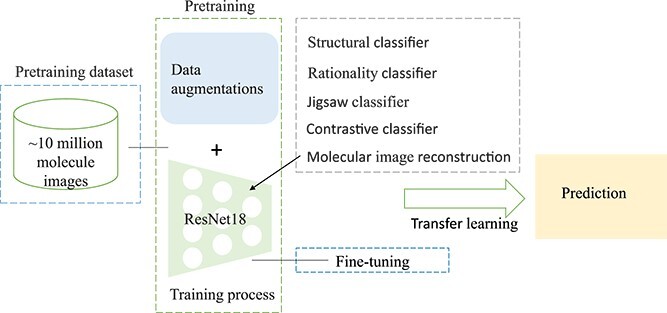
Illustration of transfer learning based molecular recognition. Instead of using labeled data, we resort to a large pretraining dataset, which is used to generate a pre-trained model and we use this model to assist the model learning. Here the pre-trained model can be equipped with various classifiers for different target tasks.

In the medical domain, acquiring extensive labeled datasets for training deep learning models is typically challenging due to the requirement for expert annotations. Transfer learning offers a solution to this constraint. In this context, pretrained CNNs trained on expansive general-purpose image datasets (such as ImageNet) can be considered as feature extractors. By transferring insights from broad image datasets to specific medical domains, the model can adeptly learn pertinent features despite the limitations of available medical data. As shown in Fig. 7, Dalkiran *et al*. [101] proposed to employ transfer learning to recognize molecular images. Li *et al*. [74] and ImageCLEF2018 [102] also utilize unsupervised or self-supervised pre-trained models to achieve transfer learning.

**Discussion.** To address the issue of limited data in drug development, transfer learning has proven to be highly effective. It leverages pre-trained models from related tasks or domains, making efficient use of existing knowledge and reducing the need for large amounts of labeled data specific to drug development. This helps overcome the challenge of data scarcity. Additionally, starting with a pre-trained model reduces training time compared with training a model from scratch, accelerating the development process. Pre-trained models can learn robust and generalizable features, leading to better performance when fine-tuned for specific drug development tasks. However, if the source and target domains are significantly different, the features learned during pre-training may not transfer well, resulting in suboptimal performance. While transfer learning reduces training time for specific tasks, the initial pre-training phase can be computationally expensive and resource-intensive. Fine-tuning pre-trained models on small datasets also carries the risk of overfitting. Furthermore, these models can be complex and difficult to interpret. Although many pre-trained models are available for common tasks like image recognition and NLP, there are fewer high-quality pre-trained models specifically tailored for drug development, limiting the applicability of transfer learning in this field.

## Applications

**Figure 8:**
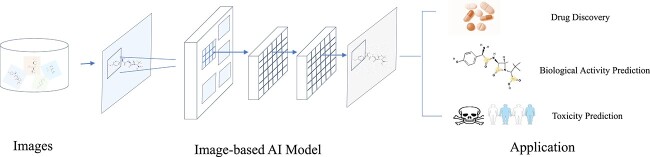
Image-based applications in drug development. Given a molecular image, we leverage CV techniques to generate visual representation, which can be applied for drug discovery, biological activity prediction, and toxicity prediction.

In this section, we provide an overview of some representative applications with the help of the image-based molecular representation learning (shown in Fig. 8). 

### Drug discovery

In recent years, the number of drugs approved through phenotypic screening has exceeded those discovered through the traditional molecular target-based approach. As a result, the predominant drug approval process now often begins with phenotypic screening, followed by an in-depth exploration of mechanisms of action and molecular targets. This paradigm shift underscores the growing importance and effectiveness of phenotypic screening in contemporary drug discovery.

There has been a significant shift in drug discovery methodologies, with researchers increasingly opting to utilize molecular drug structures directly as features, departing from the traditional reliance on open-source software for feature extraction. This transformation is grounded in the central concept of molecular targets, as discussed by Vincent *et al*. [1]. This shift has facilitated drug discovery through automated predictions of new drug–target interactions, replacing laborious and resource-intensive screening processes. The critical step in this process is identifying the physical interactions between candidate drug compounds and target biomolecules, albeit with inherent challenges in therapeutic target validation. Molecules can be represented directly through images, showcasing the molecular structure, such as MolPMoFiT and ImageMol, which are a more straightforward, direct and efficient approach compared with traditional feature extraction methods. 

### Biological activity prediction

Biological activity prediction, a crucial facet of computational chemistry, seeks to discern the intricate relationship between a molecule’s structural attributes and its ensuing biological or chemical activities. This pursuit is achieved through the mathematical or computational representation of chemical structures, followed by the application of statistical or machine learning algorithms to model and prognosticate the interplay between molecular architecture and functional outcomes.

Traditionally, QSAR models have served as the bedrock for such predictions [86, 103, 104]. These models hinge on molecular descriptors, encompassing parameters like molecular weight, electron affinity and spatial configuration, to capture the essence of molecular structure. However, an emerging paradigm shift in this field explores the utilization of molecular images for the representation of chemical entities, demonstrating the versatility of image-based molecular representation in constructing robust QSAR models, such as Chemception and QSAR-CNN.

### Toxicity prediction

Drug toxicity is a complex process that affects the human body in various ways, including liver toxicity [79], which is associated with compound components, individual factors, disease status and more. Despite this complexity, computational methods can help identify compounds that may have adverse effects on the liver early on, enabling early recognition and prevention. Traditional machine learning algorithms, such as Naïve Bayes, SVM and Random Forest, have been employed in toxicity prediction. However, as the volume of available data increases, the performance of these classic algorithms often reaches a plateau, limiting their ability to fully capture the intricacies of drug toxicity. In recent years, deep learning algorithms have emerged as a powerful approach to address the challenges of drug toxicity prediction. Deep learning methods, especially CNNs, have demonstrated consistent and remarkable improvements in drug toxicity prediction tasks. These neural networks can automatically learn intricate patterns and relationships from large-scale image molecular datasets, allowing for a more comprehensive understanding of the factors leading to drug toxicity, such as Resnet18DNN and KekuleScope.

## Limitations

### Image transformation loss

Starting from the molecular visual representation of drugs rather than drug formulations has advantages on avoiding the complex processing of drug diversity. However, the accuracy performance may be affected because information distortion occurs when mapping drug structures to images, which we call *image transformation loss*. Besides, the lack of optimization for the sparsity of molecular images can affect the extraction quality of potential features by the model, failing to acquire 3D structural information about ligands, receptors and ligand–receptor interactions. In the future, it is necessary to develop various approaches that combine image learning and other multi-view learning methods, such that we can combine information from different modalities. 

### Model generalization

Although deep learning models have achieved state-of-the-art results in various molecular property/activity prediction tasks, these end-to-end models require a large amount of training data to learn useful feature representations. The learned representations are typically specific to the existing endpoints, meaning that new endpoints or interest data sets require building and retraining models from scratch. Domain generalization methods in deep learning aim to address the challenge of model generalization across different domains or data distributions. These methods focus on learning robust feature representations that are not specific to a particular domain, enabling the model to perform well in unseen or new domains without the need for complete retraining. In the future, domain generalization methods such as Domain Adversarial Neural Networks (DANN) [105] and Gradient-Based Domain Generalization (Grad-Domain) [106] can be potentially applied to further improve the generalization. 

### Representation privacy

In addition to publicly available pharmaceutical datasets, the process of drug development involves the use of wet laboratories and patient data, which significantly increases the risk of privacy leakage when processed centrally. Federated learning [107–109] can address this issue by eliminating the need to transfer raw data to a central server. It ensures data privacy by keeping sensitive information locally on each device, thereby reducing the risk of privacy leakage. In this paradigm, models are trained locally on individual devices and only model updates are shared with the central server for aggregation. As a result, the central server never directly accesses the raw patient data, effectively protecting their privacy. Federated learning offers benefits beyond privacy. By utilizing local resources, it reduces the need for data transfer, optimizing bandwidth and mitigating network congestion. It also enables training on diverse datasets, enhancing the overall model’s performance by capturing variations across different devices and environments. In addition, encryption techniques, such as differential privacy [19], can be used to further safeguard sensitive information. In a word, optimizing the representation in a federated manner is a promising direction to explore. 

### Representation interpretability

Considering the limited physicochemical information that can be directly obtained from molecular images, it is important to enhance the representation interpretability, whose goal is to understand the reasons behind the model’s predictions for image representation. In the field of image interpretability, various methods have been proposed to understand deep learning model decision-making [110, 111]. For example, Class Activation Mapping (CAM) [112] highlights influential regions by mapping class-specific activations to the input image. Grad-CAM [113] extends CAM using gradients of the target class with respect to feature maps. Local Interpretable Model-Agnostic Explanations [114] explains predictions from any black-box model by approximating its behavior within a local neighborhood of the image. These methods enhance the understanding of deep learning decisions in images, facilitating obtaining a trust and fair representation.

## Conclusion

This paper provides the first comprehensive survey on CV- based molecular representation learning for drug development, where we investigate a large number of research papers to summarize and catch the recent advance in this field. Specifically, we propose a new taxonomy in terms of how to apply various image-based learning paradigms to assist drug development. Despite its effectiveness, we would like to highlight that the molecular visual representation has some limitations and is still in its early stage, which motivates researchers to conduct further exploration to improve its deficiency. We hope our survey could shed light on this promising field and benefit subsequent research of drug development.

**Key Points**

- We review current literature regarding the use of image-based molecular representation learning for drug development.
- We list the commonly used datasets in this field and describe the typical pipeline of molecule visual representation.
- A novel taxonomy based on deep learning paradigms is proposed.
- Potential image-empowered applications are summarized, including but not limited to drug discovery, prediction of molecular activity and toxicity assessment.
- The molecular visual representation is still in its early stage and holds significant potential. There are many areas that remain to be studied and explored in the future.

## Author Biographies

**Yue Li** is currently pursuing the M.D. degree in BUCM.

**Bingyan Liu** is a research associate professor at the School of Computer Science, BUPT.

**Jingyan Den** is currently pursuing the M.D. degree in BUCM.

**Yi Guo** is the attending physician of the Gastroenterology Department at Dongzhimen Hospital, BUCM.

**Hongbo Du** is the Chief Physician of the Gastroenterology Department at Dongzhimen Hospital, BUCM.
